# PD-L1 Up-Regulation in Prostate Cancer Cells by *Porphyromonas gingivalis*

**DOI:** 10.3389/fcimb.2022.935806

**Published:** 2022-06-29

**Authors:** Sabine Groeger, Fan Wu, Florian Wagenlehner, Temuujin Dansranjav, Sabine Ruf, Fabian Denter, Joerg Meyle

**Affiliations:** ^1^Dept. of Periodontology, Justus-Liebig University, Giessen, Germany; ^2^Dept. of Orthodontics, Justus-Liebig University, Giessen, Germany; ^3^Clinic of Urology, Justus-Liebig University, Giessen, Germany

**Keywords:** periodontitis, prostate cancer, *Porphyromonas gingivalis*, PD-L1 up-regulation, immune evasion

## Abstract

Chronic inflammation is known to contribute to various human cancers. *Porphyromonas gingivalis* (*P. gingivalis*), is a gram-negative oral keystone pathogen that may cause severe periodontitis and expresses several virulence factors to affect the host immune system. Periodontitis is a chronic infectious disease that while progression, may cause loss of attachment and destruction of the tooth supporting tissues. Prostate cancer is one of the most common malignancies in men. Increasing evidence links periodontitis with prostate cancer, however the mechanisms explaining this relationship remain unclear. The aim of this study was to investigate the expression and signaling pathway of programmed death ligand 1 (PD-L1) in a prostate cancer cell line after infection with *P*. *gingivalis* and stimulation with *P*. *gingivalis* components to reveal the mechanism of tumor-induced immune evasion associated with bacterial infection in the tumor environment. Prostate cancer cells were infected with different concentrations of viable *P. gingivalis* and treated with different concentrations of heat-killed *P. gingivalis* and *P. gingivalis* cell components, including the total membrane fraction, inner membrane fraction, outer membrane fraction, cytosolic fraction and peptidoglycan (PGN). Chemical inhibitors were used to block different important molecules of signaling pathways to assess the participating signal transduction mechanisms. PD-L1 expression was detected by Western blot after 24 h of infection. PD-L1 was demonstrated to be upregulated in prostate cancer cells after infection with viable and with heat-killed *P. gingivalis* membrane fractions. Also isolated PGN induced PD-L1 up-regulation. The upregulation was mediated by the NOD1/NOD2 signaling pathway. No upregulation could be detected after treatment of the cells with *P. gingivalis* lipopolysaccharide (LPS). These results indicate, that chronic inflammatory disease can contribute to tumor immune evasion by modifying the tumor microenvironment. Thus, chronic infection possibly plays an essential role in the immune response and may promote the development and progression of prostate cancer.

## Introduction

Periodontitis is a chronic inflammation of the periodontium caused by oral microbes that leads to destructive processes in the periodontal supporting tissues and eventually tooth loss. Evidence indicates a link between periodontitis and systemic illness such as cardiovascular diseases, diabetes, adverse pregnancy outcomes and rheumatoid arthritis (RA) (Cullinan et al., 2009). A correlation between periodontitis and cancer is also established; periodontitis is most consistently linked to increased risks of oral and esophageal cancers (Fitzpatrick and Katz, 2010), but it is also associated with increased risks of other cancer types, such as prostate cancer (Fitzpatrick and Katz, 2010; Lee et al., 2017b; Guo et al, 2021; Wei et al, 2021). Among the numerous bacterial species that exist in the oral cavity, *Porphyromonas gingivalis* (*P. gingivalis*) is associated with severe forms of periodontitis (Mysak et al., 2014). *P. gingivalis* a gram-negative, anaerobic oral periodontopathogen is considered as a keystone pathogen, which enables the proliferation of other subgingival bacteria and initiates host inflammatory responses; thus, it is strongly correlated with periodontitis (Hajishengallis et al., 2012).

*P. gingivalis* membranes exhibit bioactive components typical for gram-negative bacteria, including the inner membrane (IM), a thin peptidoglycan (PGN) layer, and OM. The cellular membrane of *P. gingivalis* is essential for protection of the bacteria and for the transfer of a variety of proteins to the infectious environment (Bos et al., 2007).

PGN shields bacteria from the host´s enzymes and enables bacterial survival within the host cells (Wang et al., 2012). OMVs are enriched with proteins originating from the OM, LPS, cytosol and PGN and they are one way for *P. gingivalis* to release virulence factors into the environment (Veith et al., 2014).

Globally prostate cancer is the second most frequently diagnosed malignancy in men and is the second most common cause of mortality. There are an estimated 137.9 new cases per 100,000 men every year, with approximately 1.3 million new cases and 359,000 associated deaths worldwide in 2018 and estimated that 385,560 deaths globally in 2020 (Ferlay et al., 2015; Siegel et al., 2015; Siegel et al., 2016; Bray et al., 2018).

Inflammation is regarded as a primary risk factor for a variety of cancers. Studies revealed that nearly 15% of tumors worldwide are linked to microbial infection (Kuper et al., 2000).

Infection and inflammatory environments have been demonstrated to speed up prostate cancer progression in human as well as in animal studies. The mechanisms how infectious agents contribute to prostate carcinogenesis are poorly understood and remain to be further investigated (Sutcliffe and Platz, 2008; Jiang et al., 2013; Sfanos et al., 2013). A number of pathogens are able to induce prostatitis, including viruses, fungi, bacteria and parasites (Nakai and Nonomura, 2013). The most commonly identified bacterium leading to prostatitis is the gram-negative bacterium *E. coli* (Domingue and Hellstrom, 1998). SActivation of TLRs on the cell surface and cytosolic nucleotide-binding and oligomerization domain (NOD) receptors, which play a major role in sensing bacterial components, also contribute to cancer progression (Kang et al., 2012; Moreira and Zamboni, 2012; Sfanos and De Marzo, 2012).

The oral microbiome has been shown to be associated with prostate infection as well. Oral bacteria such as *P. gingivalis* and *T. denticola* have been detected in prostatic secretions and dental plaques from patients suffering from both, prostatitis and periodontitis (Estemalik et al., 2017).

PD-L1 (also known as B7 homolog 1 (B7-H1) or cluster of differentiation (CD) 274) is a ligand of PD-1. Binding of PD-L1 to PD-1 modulates the immune response. The PD-1/PD-L1 axis is item of broad investigation in cancer immunotherapeutic research. In health, the PD-1/PD-L1 pathway maintains homeostasis in immune reaction, while in a state of microbial infection it shelters the host from hyper-activated T effector cells (TEFFs). These hyper-activated TEFFs can support autoimmune diseases or chronic infections (Sharpe et al., 2007). PD-L1 expression is linked to tumor aggressiveness and chronic infections (Boussiotis, 2016).

*P. gingivalis* total membrane was shown to activate the interleukin (IL)-1 receptor-associated kinase (IRAK)/NF-kB pathway (Groeger et al., 2017b). Besides these main signaling pathways, other pathways are involved in PD-L1 regulation. After recognition of bacterial molecules by NOD1 and NOD2 a threonine-protein kinase 2 (RIP2) kinase (also known as threonine kinase (RICK)) is activated which transduces signals by MAPKs or the transcription factor NF-kB. This process results in the initiation of immune response by inflammatory cytokine genes, or by c-Jun N-terminal kinase (JNK) and activates the transcription factor AP-1 (Park et al., 2007; Hewitt et al., 2012; Caruso et al., 2014; Groeger et al., 2017b).

PD-L1 expression can be altered by bacterial infection. *H. pylori* and *P. gingivalis* both can induce PD-L1 expression in different cell lines (Silva et al., 2016; Groeger et al., 2017c). Viable or heat-killed *P. gingivalis*, as well as membranes of *P. gingivalis* induce PD-L1 expression both in cancer and in other epithelial cell lines (Groeger et al., 2017a; Groeger et al., 2017c). Low multiplicity *P. gingivalis* infection for 5–23 weeks was demonstrated to induce PD-L1 expression in human immortalized oral epithelial cells (Lee et al., 2017a).

The aim of this study was to investigate the impact of the periodontopathogenic bacterium *P. gingivalis* on the expression of immune checkpoint programmed death-ligand 1 (PD-L1) on prostate cancer cells. The hypothesis, that components of *P. gingivalis* activate mechanism of infection-induced immune evasion in the tumor environment, should furthermore be assessed.

## Materials and Methods

### Cell Cultures

The human prostate cancer cell line DU-145 was purchased from Leibniz-Institut DSMZ-Deutsche Sammlung von Mikroorganismen und Zellkulturen GmbH (DSMZ), DSMZ no. ACC 261. Cells were cultured in a medium containing Dulbecco’s Modified Eagle Medium (DMEM): Ham’s F12(4:1, vol:vol), 10mM HEPES (Invitrogen, Karlsruhe, Germany) and 10% fetal calf serum (FCS), Greiner, Frickenhausen, Germany). The cells were seeded in 6-well plates, 1x10^6^ cells per well and grown at 37°C in a humidified atmosphere with 5% CO_2_ to 80% confluency before stimulation.

### Bacterial Growth

*Porphyromonas gingivalis* W83 was purchased from the American Type Culture Collection (ATCC, LGC Standards GmbH, Wesel, Germany). It was grown at 37°C in brain-heart-infusion broth (Difco, BD, Heidelberg, Germany) with hemine (5 µg/ml) and menadione (1 µg/ml) (Sigma-Aldrich, Munich, Germany) under anaerobic conditions using the Anaerogen system (ThermoFisher, Dreieich, Germany) until late log phase (OD=1).

### Chemicals and Stimulants

Lauroyl-g-D-glutamyl-meso-diaminopimelic acid (C12-iE-DAP) (Invivogen #tlrl-c12dap), an acylated derivate of the peptidoglycan-like dipeptide, was used to study the role of peptidoglycan in the PD-L1 expression in epithelial cells. C12-iE-DAP is a NOD agonist that activates intracellular receptor NOD1 and leads to NF-kB activation. *P. gingivalis* TM (70 µg/ml) was used as a positive control.

IFN-γ (Miltenyi Biotec) was used in doses of 100–1000 U/ml to stimulate the prostate cancer cells. Cells were incubated for 24h. The NOD1 ligand -d-Glu-mDAP (C12-iE-DAP, 100 g/ml, Invivogen) was used to study the role of peptidoglycan in the PD-L1 upregulation in prostate cancer cells and if the NOD1/NOD2 signaling pathway is involved.

*P. gingivalis* W83 LPS was extracted with hot phenol/water and purified by ultracentrifugation and enzymatic treatments as described (De Castro et al., 2010). The lyophilized LPS was re-suspended in endotoxin-free water to obtain a stock solution with a concentration of 10 mg/ml. *E. coli* 055:B5 LPS was purchased from Sigma-Aldrich and re- suspended in endotoxin-free water to obtain a stock solution of 1 mg/ml.

### Preparation of Bacterial Fractions

Bacteria were harvested in the late exponential growth phase (OD600 of 1.0) by centrifugation for 20 min at 6500 × g at 25°C. The pellet was re-suspended in 50 ml of 10 mM HEPES, pH 7.4, containing a protease inhibitor cocktail (4 mini- tablets of Complete, EDTA-free, Roche) and DNase I/RNase A (20 μg/ml each).

Bacteria were disrupted by four passages through a high-pressure cell disruption system (Model TS, 0.75KW, Constant Systems Ltd.) at 40,000 psi. The cellular debris was removed by centrifugation at 8,000 × g for 30 min at 4°C. The membranes were sedimented from the cleared lysate at 150,000 x g for 2 h at 4°C. The supernatant (cytosolic fraction) was stored, and the total membrane fraction was washed three times with 10 mM HEPES, pH 7.4. The membrane pellet was subsequently re-suspended in 10 mM HEPES, pH 7.4, and layered onto a discontinuous sucrose gradient to separate the total membranes into the outer and inner membranes by ultracentrifugation at 96,808 × g for 20 h at 4°C as described (Schnaitman, 1970; Koplow and Goldfine, 1974). Fractions were assayed for protein content (Bio-Rad Protein Assay Reagent), and the inner and outer membrane fractions were pooled, diluted with 10mM HEPES, pH 7.4 and then sedimented by ultracentrifugation at 150,000 × g for 2 h at 4°C. The protein concentrations of all samples, cytosolic fraction, total membranes and outer membrane fractions, were determined using Bio-Rad’s protein assay reagent. The purity of the fractions was confirmed by sodium dodecyl sulfate polyacrylamide gel electrophoresis (SDS PAGE). For the stimulation experiments *in vitro*, oral epithelial cells were co-incubated with various concentrations (10 μg/ml − 100 μg/ml) of the isolated fractions in a dose- and time-dependent manner.

### Inhibition of Signaling Pathways

The c-Jun N-terminal kinases (JNK) inhibitor SP00125 was used in 10, 50 and 100μM concentrations, the receptor-interacting serine/threonine-protein kinase 2 (RIP2) inhibitor Gefitinib was added in 2.5 µM and 10 µM concentrations following the manufacturer´s recommendations. All inhibitors (Invivogen, Toulouse, France) were pre-incubated for 1h before stimulation with membrane fraction for another 24h.

### Isolation of Peptidoglycan

Peptidoglycan of *P. gingivalis* was isolated according to a protocol of Desmarais (Desmarais et al., 2014) with modifications. A late logarithmic culture of *P. gingivalis* was centrifuged at 8.000 x g for 10 minutes. Pellet was resuspended and trichloric acid (10%) was added and incubated for 30 minutes at 4°C. Cells were washed three times in phosphate-buffered saline (PBS) and after last washing slowly pipetted into boiling sodium dodecyl sulfate (SDS) (final concentration 4%). Cells were boiled for 3 hours and continued stirring overnight without heat. Samples were centrifuged at 150.000 x g for 60 minutes at room temperature to pellet the peptidoglycan polymers. Pellet was resuspended in water and washed three times to remove remaining SDS, and resuspended in 10 mM Tris-HCl. Proteinase K digestion was performed and samples were spun down by 150.000 x g for 60 minutes at room temperature, weighted and resuspended in water.

### Infection of Prostate Cancer Cells With *P. gingivalis* W83

For bacterial infection of 1 × 10^6^ prostate cancer cells, *P. gingivalis* was harvested in the late exponential growth phase (OD_600_ of 1.0) by centrifugation, washed and resuspended in an appropriated volume of cell culture medium to achieve the desired cell-bacteria ratio. The bacterial cell number was adjusted based on spectrophotometric measurements of the optical density of the bacterial suspension at 600 nm (OD_1_ = 10^9^ cells/ml).

### Heat-Killed Bacteria

*P*. *gingivalis* culture was harvested and washed in PBS. Cells were heat-killed at 70°C for 10 minutes. Heat-killed bacteria were checked by plating cells on Brucella agar plates in 37°C (Thermo Fisher, Dreieich, Germany). Bacterial suspensions were adjusted in a concentration of 10^9^ cells/ml using optical density measurements and the multiplicity of infection (MOI) 10, 100 and 500 was used for stimulation (ThermoFisher BSA Protein Assay Kit).

### Western Blot

To investigate PD-L1 expression upon infection with *P*. *gingivalis*, Western blot analysis was performed. Cells were stimulated with *P*. *gingivalis* fractions in different concentrations in a dose- and time-dependent manner. 1 x 10^6^ cells were seeded per well in a 6-well plate and incubated until adherence of epithelial cells for up to 2 hours. Inhibitors were added to the medium and cells were incubated for one additional hour before stimulation or infection. After 24h cells were washed, harvested and lysed using RIPA extraction and lysis buffer (Thermo Fisher). Normalization was achieved by measuring concentrations of the samples and by Ponceau S stain (Aldridge et al., 2008; Romero-Calvo et al., 2010). Protein concentrations were determined by BCA-assay and equal amounts of protein (20µg) were loaded on SDS gel. After protein separation, the proteins were transferred on nitrocellulose membranes by semi-dry transfer (BioRad Turboblotting) for 7 minutes (constant 1 A, max 25V). For antigen detection, membranes were blocked in 2% milk powder in TBST buffer for 1 hour and then incubated in primary antibody (Rabbit Anti-human PD-L1, Thermo Fisher #PA-5-20343 for 1: 2000 dilution, NOD1 Thermo Fisher #PA-5-18027 for 1:1000 dilution, NOD2 Thermo Fisher #PA-5-18572 for 1:000 dilution in 2% milk powder in TBST buffer) overnight at 4°C. Washing with TBST for 10 minutes was performed 3 times, then membranes were incubated in the secondary antibody (Goat Anti-Rabbit, Thermo Fisher #32460, 1:500 in 2% milk powder in TBST buffer) 1 hours at room temperature. Antibody specificity for PD-L1 was checked using a blocking peptide (BP) (Thermo #PEP-0463) in a pre-adsorption assay. Following three washing steps, the blots were incubated in enhanced chemiluminescence (ECL) reagent (Bio-Rad Max). Chemiluminescence was detected using X-ray films (Kodak). X-ray films were scanned and the band intensities were measured by ImageJ software (SciJava).

### Quantitative Real Time Polymerase Chain Reaction (PCR)

The cells were harvested after 24h for ribonucleic acid (RNA) extraction. The RNA was extracted using the Nucleo Spin^®^ RNA Plus columns and solutions following the manufacturer´s instructions (Machery-Nagel, Dueren, Germany). Reverse transcription was performed with the Verso cDNA sythesis kit (ThermoFisher) following the manufacturer´s protocol. For quantitative real time (RT) PCR the SensiFAST ™SYBR No-ROX Mix was used (Bio Cat, Heiderberg, Germany). As house-keeping reference gene glycerinaldehyd-3-phosphat-dehydrogenase (GAPDH) was used. As primer the Quantitect Primer assay Hs_CD274_1_SG (PD-L1), Hs_RIPK1_1_SG (RIP2) and Hs_GAPDH_1_SG (GAPDH) were chosen (Qiagen, Hilden, Germany).

PCR runs and detection were performed in a BioRad CFX96 Real-Time System C1000 Thermal Cycler (BioRad, Feldkirchen, Germany).

The outcome was analyzed using the ΔΔ CT method. The results are shown normalized to reference and relative to non-treated negative control

### Statistical Analysis

All experiments were repeated at least in three independent experiments. The results were analyzed using independent two-sample Student’s t-test and corrected for multiple comparison using the Bonferroni-Holm correction. The character of the evaluation was explorative. Probability of error was set to 5% and shown as p-values.

### Ethical Considerations

All experiments followed the guidelines of good clinical/laboratory practise (GCP/GLP) and the WHO declaration (Helsinki, 1964), latest update Seoul 2008 (59th WMA General Assembly, Seoul, October 2008).

### Additional Information

The authors declare no conflict of interests.

## Results

### PD-L1 Expression Is Up-Regulated by Viable and Heat-Killed *P. gingivalis* W83

**Figure 1:**
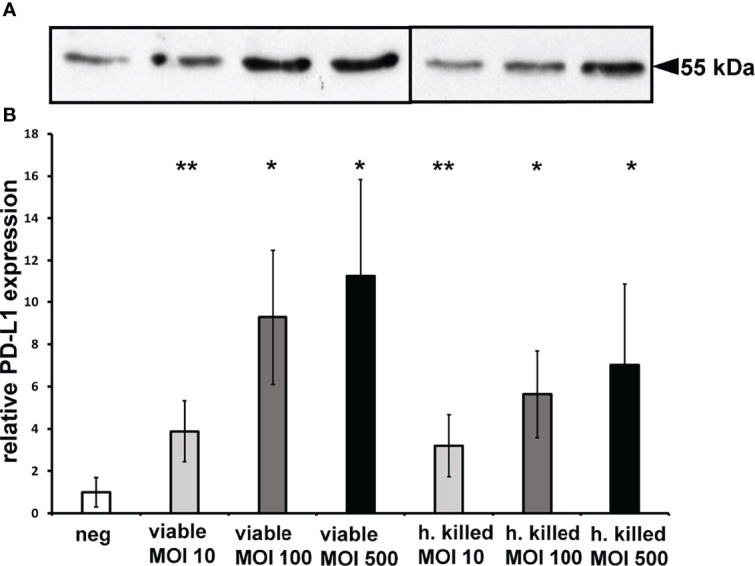
Heat-killed and viable bacteria induce PD-L1 expression: **(A)** Exemplary Western blot of PD-L1 expression in DU-145 cells after 24h stimulation with viable or heat-killed *P. gingivalis* W83. **(B)**: Western blot of PD-L1 mean expression in DU-145 cells after 24h stimulation with *P. gingivalis* W83; neg, non stimulated cells; v, viable bacterial infection; h, heat-killed bacterial infection; MOI, multiplicity of infection (n=3); * indicates corrected p < 0.05, ** indicates corrected p < 0.01.

PD-L1 expression was analyzed 24h after *P. gingivalis* infection of the prostate cancer cell line DU-145. Infection with viable *P. gingivalis* caused 3.8 ( ± 1.7) fold up-regulation of PD-L1 expression at multiplicity of infection (MOI) of 10, increased to 9.3 ( ± 3.9) fold using MOI 100 and 11.3 ( ± 5.5) fold using MOI 500 (**Figure 1**). Infection with heat-killed *P. gingivalis* caused 4-fold of PD-L1 expression at MOI 10 and MOI 100 and 7-fold at MOI 500 (**Figure 1**). **Figure 1A** shows an exemplary western blot, 1B the results of the analysis of at least 3 experiments, n = 3, * indicates corrected p < 0.05, ** indicates corrected p < 0.01 compared to control.

### PD-L1 Expression Is Up-Regulated *by P. gingivalis* Fractions

**Figure 2:**
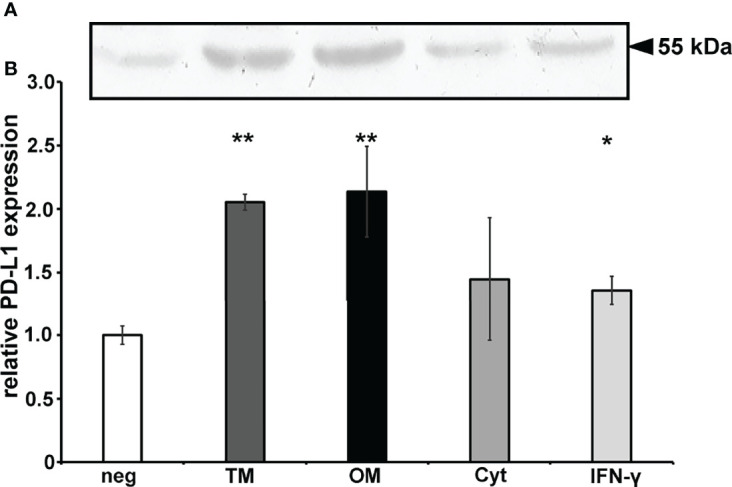
PD-L1 expression is up-regulated by *P. gingivalis* fractions: **(A)** Western blot of PD-L1 expression in DU-145 cells after 24h stimulation with viable or heat-killed *P. gingivalis* W83 as well as its fractions. IFN-γ is used as a positive control. **(B)** *P. gingivalis* induced expression of PD-L1 on DU-145 cells after 24 h stimulation with different fractions of *P. gingivalis* W83. IFN-γ is used as a positive control. Neg, non stimulated cells; TM, total membrane; OM, outer membrane; Cyt, cytosolic fraction. (n=3), * indicates corrected p < 0.05, ** indicates corrected p < 0.01 compared to control.

PD-L1 expression was analyzed after infection with *P. gingivalis* fractions of the prostate cancer cell line DU-145 after 24h, using IFN-γ as positive control (**Figure 2**). The results demonstrated that *P. gingivalis* membrane fractions induced up-regulation of PD-L1 expression in prostate cancer cell line DU-145. Total membrane fractions and outer membrane fractions of *P. gingivalis* increased PD-L1 expression 3.3 ( ± 0.5) and 3.4 ( ± 0.5) fold, cytosolic fractions and IFN-γ increased PD-L1 expression 2.3 ( ± 0.4) and 2.2 ( ± 0.5)-fold.

**Figure 2A** shows an exemplary western blot, 2B the results of the analysis of at least 3 experiments, n = 3, * indicates corrected p < 0.05, ** indicates corrected p < 0.01 compared to control.

### Chemical Inhibition of *P. gingivalis* Membrane Induced PD-L1 Expression

**Figure 3:**
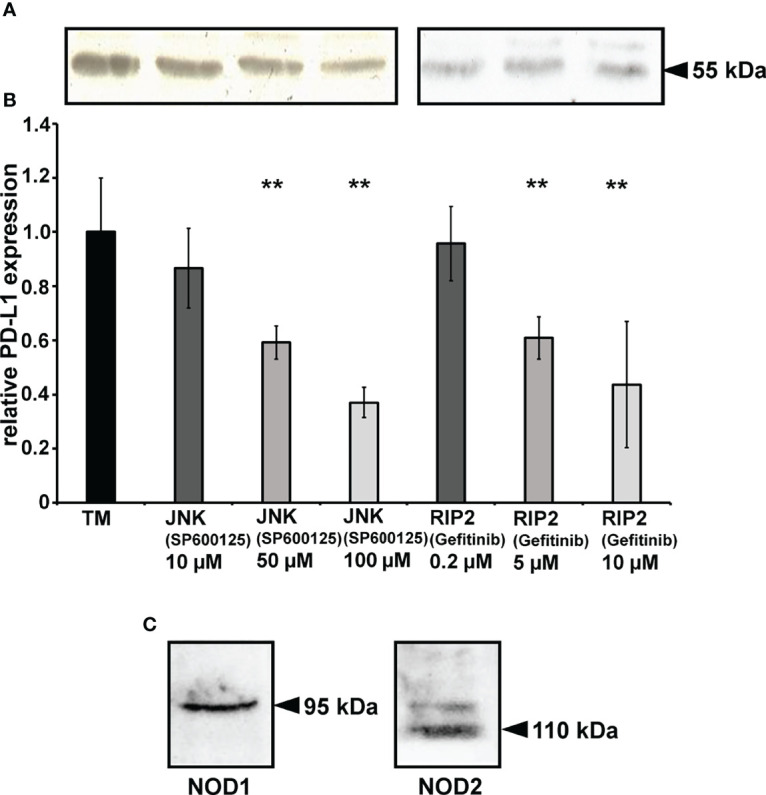
Chemical inhibition of PD-L1 expression: **(A)** Western blot of Signaling pathway of PD-L1 expression was investigated by SP00125, a specific inhibitor of JNK (MAP kinase pathway) and Gefitinib an RIP2 (NOD pathway) inhibitor. **(B)** Signaling pathway of *P. gingivalis* induced PD-L1 expression was investigated using SP00125, a specific inhibitor of JNK (MAP kinase pathway) and Gefitinib an RIP2 (NOD pathway) inhibitor. TM = total membrane. (n=3), ** indicates corrected p < 0.01 compared to control. **(C)** The prostate cancer cell line DU-145 expresses NOD1 and NOD2 receptors. NOD1/NOD2 receptors protein expression in prostate cancer cell line DU145 was investigated using Western blot (n=3).

*P. gingivalis* total membrane induced PD-L1 expression was modified using inhibitors of the JNK (SP600125) and RIP2 (Gefitinib) pathway. PD-L1 expression was analyzed by Western blot after infection with *P. gingivalis* total membranes alone or with chemical inhibitors in the prostate cancer cell line DU-145 after 24h (**Figures 3A, B**). In comparison to the stimulation with TM (70µg/ml) after 24h, 10µM SP600125 decreased the expression to 86.6% (± 15), 50 µM to 60.7% (± 8) and 100 µM to 37% (± 6). Gefitinib (2.5 µM) reduced the expression to 95.7% (± 14), 5 µM to 60.9% (± 8) and 10 µM to 62.7% (± 3). **Figure 3A** shows an exemplary western blot, B the results of the analysis of at least 3 experiments, n = 3, * indicates corrected p < 0.05, ** indicates corrected p < 0.01 compared to control.

Western Blot analysis revealed, that both NOD receptors, NOD1 and NOD2, are expressed constitutively in DU-145 cells. (**Figure 3C**).

### PD-L1 Protein Expression Is Up-Regulated by the NOD Agonist C12-iE-DAP and *P. gingivalis* Peptidoglycan (PGN)

**Figure 4:**
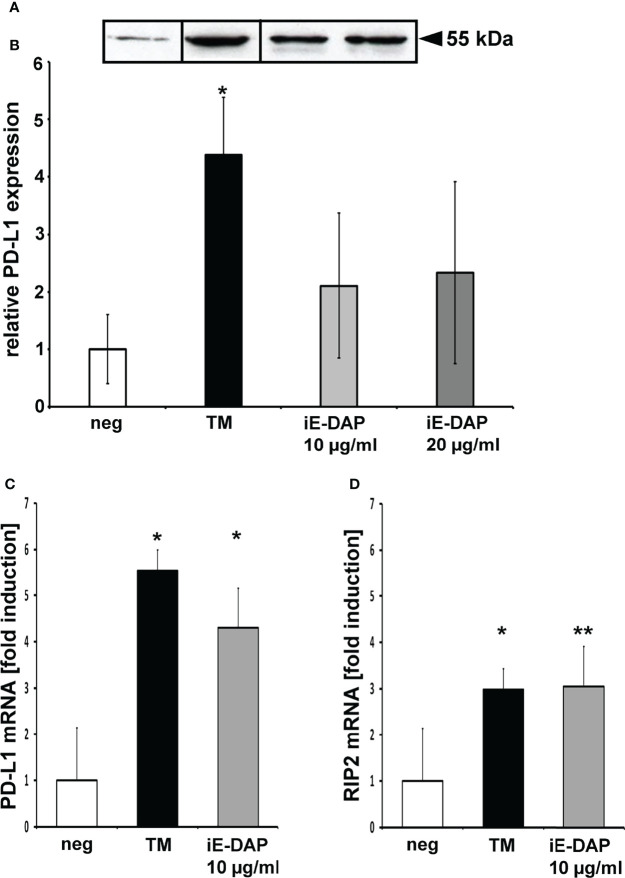
PD-L1 expression is up-regulated by peptidoglycan motifs. **(A, B)** Western blot of DU145 cells after 24h stimulation with C12-iE-DAP as a NOD1 agonist in a dose-dependent manner. **(C)** PD-L1 mRNA expression is up-regulated in DU145 cells after 24h stimulation with C12-iE-DAP as a NOD1 agonist in a dose-dependent manner. **(D)** RIP2 mRNA expression is up-regulated in DU145 cells after 24h stimulation with C12-iE-DAP in a dose-dependent manner; neg, non-stimulated cells, TM, total membrane; C12, C12-iE-DAP; **(C)** (n=3) * indicates corrected p < 0.05, ** indicates corrected p < 0.01 compared to control.

Infection with 10 µg/ml or 20 µg/ml C12-iE-DAP induced 2.1 (± 1.2) and 2.3 (± 1.5) fold PD-L1 up-regulation (**Figures 4A, B**).

QPCR revealed that TM induces the PD-L1 mRNA 5.54 (± 0.5) fold and iE-DAP 4.3 ( ± 0.9) fold (**Figure 4C**). The RIP2 mRNA expression was induced 3 ( ± 0.5) fold by TM and also 3 ( ± 0.4) fold by iE-DAP (**Figure 4D**).

**Figure 4A** shows an exemplary western blot, 4B the results of the analysis of at least 3 experiments, n = 3, * indicates corrected p < 0.05, ** indicates corrected p < 0.01 compared to control.

### PD-L1 Protein Expression Is Up-Regulated by Isolated *P. gingivalis* Peptidoglycan But Not by *P. gingivalis* or *E. coli* LPS

**Figure 5:**
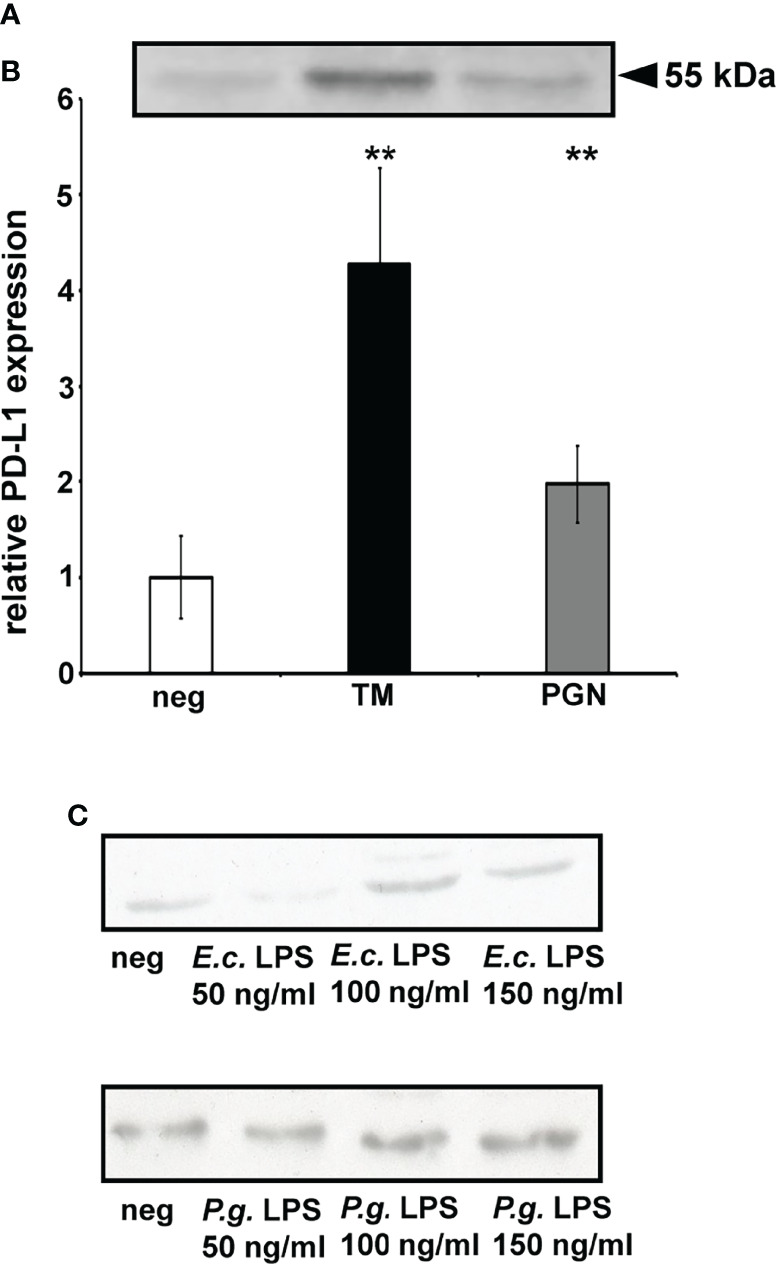
PD-L1 expression is up-regulated by *P. gingivalis* peptidoglycan (PGN) but not LPS. **(A)** Western blot of PD-L1 expressed by DU145 cells after 24h stimulation *P. gingivalis* isolated PGN. **(B)** PD-L1 expression is up-regulated in DU145 cells after 24h stimulation *P. gingivalis* isolated PGN. (n>3) ( **= p < 0.01.), neg = non- stimulated cells, TM, total membrane, peptidoglycan = 10 μL isolated *P. gingivalis* peptidoglycan. **(C)** No up-regulated expression was detected after stimulation with *P. gingivalis* LPS or *E. coli* LPS. (n=3). Neg = non-stimulated cells, *E. coli* and *P. gingivalis* LPS stimulation in a dose-dependent manner. ** indicates corrected p < 0.01 compared to control.

Data showed that up-regulation of PD-L1 with 10 µL isolated *P. gingivalis* peptidoglycan simulation is 2.0 (± 0.4) fold (**Figures 5A, B**).

To investigate if TLRs are involved in PD-L1 up-regulation, DU-145 cells were stimulated with *E. coli* and *P. gingivalis* LPS. Neither *E. coli* nor *P. gingivalis* LPS induced PD-L1 expression in prostate cancer cell line (**Figure 5C**); n = 3, * indicates corrected p < 0.05, ** indicates corrected p < 0.01 compared to control.

## Discussion

This study investigated the expression and signaling pathway of PD-L1 in prostate cancer cells after infection with *P. gingivalis.* The effects of *P. gingivalis* membrane fractions and isolated PGN on a prostate cancer cell line revealed the mechanisms of tumor-induced immune evasion, which corresponds with bacterial infection in tumors and tumor microenvironment. The results showed that infection with *P. gingivalis* and its components up-regulated PD-L1 expression in prostate cancer cells. Groeger et al., 2020 showed the same mechanism in oral cancer cells and found that the bacterial cell wall component PGN triggers cytosolic NOD receptors to induce PD-L1 expression in a RIP2-dependent manner (Groeger et al., 2020). Gram-negative bacteria are able to deliver PGN into host cells using cellular invasion or a bacterial secretion system (Girardin et al., 2001; Viala et al., 2004). Transport into cells *via* OMV transport as a generalized mechanism was also proposed whereby gram‐negative bacteria deliver PGN to cytosolic NOD1 (Kaparakis et al., 2010). In this study, the NOD1 agonist iE-DAP and PGN isolated from *P*. *gingivalis* both upregulated PD-L1 expression in prostate cancer cells, suggesting that the *P. gingivalis*-mediated induction of PD-L1 expression in prostate cancer cells is PGN-dependent.

LPS-mediated induction of PD-L1 expression has been shown in different immune cell types (Yamazaki et al., 2002; Loke and Allison, 2003; Zhang et al., 2016). This effect could not be confirmed using oral epithelial cells (Groeger et al., 2017c). It was reported that LPS induced PD-L1 expression after 4h stimulation in immune cells did not last for up to 24h (Hewitt et al., 2012). It can be presumed, that PGN or other NOD1 or NOD2 agonistic molecules are present in LPS preparations due to the process of preparation (Park et al., 2007).

NOD receptors detect bacterial PGN and are localized in the cytosol. Recognition of bacteria induces activation of the RIP2 kinase, which transduces signals over MAPKs or the transcription factor NF-kB to activate immune response genes such as inflammatory cytokines, or *via* JNK to activate the transcription factor AP1 using a MAPK pathway (Park et al., 2007; Hewitt et al., 2012; Caruso et al., 2014; Groeger et al., 2017b).

The results of the current study show that NOD1/NOD2/RIP2/JNK pathway activation is involved in the *P. gingivalis* total membrane-induced PD-L1 expression. There is evidence that the MAPK but also NF‐κB pathways is initialized. The pro-inflammatory transcription factor NF‐κB was shown to be activated by NOD1 *via* RIP2 in plasmid‐transfected epithelial cell lines (Inohara et al., 1999). Aberrant activation of NF‐κB was linked to cancer development, inflammatory response and autoimmune diseases (Groeger et al., 2017b; Asgarova et al., 2018). MAPK pathways are essential in the cell cycle but also in pro-inflammatory cytokine production. In an earlier study, it was reported that NOD1/NOD2 agonists activated the NF-κB and MAPK pathways in the prostate cell line DU-145 (Kang et al., 2012). This work demonstrated that NOD1/NOD2 agonist-mediated activation of the NF-κB and MAPK pathways was responsible for PD-L1 upregulation in the DU-145 cell line, possibly revealing the mechanisms of *P. gingivalis* impact and pathway of bacterial-induced PD-L1 upregulation.

TLRs and NODs are receptors that recognize bacterial molecular patterns. NOD1 and NOD2 are expressed in multiple cell types, but results from studies implied that NOD1 or NOD2 agonists alone do not induce cytokine production in prostate epithelial cells (Kang et al., 2012). NOD1 or NOD2 agonists can activate NF-κB and MAPK pathways together with TLR agonists, such as LPS, which subsequently induce cytokine production (Park et al., 2007; Kim et al., 2008; Kang et al., 2012). NOD1 and NOD2 can be detected in various prostate lesions, including prostatic intraepithelial neoplasia (PIN), phyllodes-like tumors, and adenocarcinoma in transgenic adenocarcinoma of the mouse prostate (TRAMP) (Kang et al., 2012). Gram-negative bacteria are able to transfer PGN into host cells using different mechanisms (Girardin et al., 2001; Viala et al., 2004). The current study demonstrated that the PD-L1 inducing component is localized in the membrane part of *P. gingivalis.*

*P. gingivalis* total membrane-induced PD-L1 upregulation is independent of myeloid differentiation primary response 88 (MyD88), which is a downstream signaling molecule of TLRs that activates the NF-κB pathway (Groeger et al., 2017b). Crosstalk between the NF-κB and NOD pathways seems to be crucial for bacterial PGN induced PD-L1 up-regulation. NOD combined with TLR activation by their respective agonists is a prerequisit for cytokine production (De Kimpe et al., 1995; Fritz et al., 2006; Kim et al., 2011; Zhang et al., 2011). *P. gingivalis* and its membrane components contain not only PGN that is a NOD agonist, but also LPS that is a TLR agonist. These agonists possibly activate the NF-κB and NOD pathways simultaneously (Fritz et al., 2006; Groeger et al., 2017b). Another study group has shown similar results using prostate epithelial cancer cells, demonstrating that NOD1 and NOD2 stimulation can activate NF-kB and MAPK (Kang et al., 2012). The results of the current study revealed that *P. gingivalis* PGN is responsible for PD-L1 upregulation, in contrast to *P. gingivalis* LPS that did not induce this effect. These data indicate that TLR activation is not responsible for the observed upregulation. Simultaneous activation of the NF-kB and MAPK pathways seems to be of importance for PD-L1 expression in prostate cancer cells.

Previous studies have demonstrated that *P*. *gingivalis* W83 upregulates PD-L1 expression in oral cancer cells and in primary as well as immortalized human gingival keratinocytes (Groeger et al., 2011). Furthermore, *P*. *gingivalis*-induced upregulation of PD-L1 expression has been demonstrated in several studies. *P*. *gingivalis* membrane fractions, OMVs and isolated PGN were proven to up-regulate PD-L1 expression on oral cancer cells, both on protein and also on mRNA levels (Groeger et al., 2017c). This actual study demonstrates, for the first time, that both viable as well as heat-killed *P. gingivalis* and its membrane fractions and PGN induce PD-L1 upregulation dose-dependently in prostate cancer cells.

PD-L1 is highly expressed in aggressive primary prostate cancer and is a prognostic marker for tumor growth and cancer progression and (Gevensleben et al., 2016). Clinical-phase therapies using antibodies with PD-L1 and its ligands as targets have shown promising response rates in various cancers. PD-L1 expression in the tumor environment is associated with postoperative recurrence and impaired prognosis in patients suffering from different cancers (Gao et al., 2009; Topalian et al., 2012). Ongoing research currently investigates the molecular mechanisms that are involved (McAllister et al., 2018). Early clinical trials revealed that mCRPC patients do not benefit from to PD-L1 blockade as much as patients suffering from other cancers, although 31.6% of mCRPC patients express the PD-L1 receptor (Goswami et al., 2016; Haffner et al., 2018). Thus, deeper understanding of the underlying mechanisms of PD-L1 expression in prostate cancer is required. This study proves that PD-L1 expression is inducible in prostate cancer cells and can be upregulated by bacterial infection. These findings support the hypothesis that immune checkpoint therapy may also be feasable in prostate cancer. Furthermore, the results imply that bacterial infection might be a risk factor for prostate cancer development. Future studies will elucidate the interactions and underlying mechanisms.

Inflammation is regarded as a critical factor for carcinogenesis since a long time. There is evidence that chronic inflammation can cause DNA damage and induces inflammatory products that contribute to tumor growth (Coussens and Werb, 2002).

Inflammation is a risk factor for the development of a multitude of cancers (Coussens and Werb, 2002). A large number of studies have provided evidence of a link between prostatitis and prostate cancer, but the underlying mechanisms are not fully understood (Roberts et al., 2004; Arora et al., 2010; Sfanos and De Marzo, 2012).

Periodontitis is a prevalent chronic inflammatory disease that is characterized by inadequate immune response in the host that eventually induces destruction of tooth-supporting tissues. *P*. *gingivalis* is regarded as keystone pathogen in a dysbiotic microbial community with the ability to provoke periodontitis.

The results of a 12-year longitudinal cohort study on a large population in South Korea has shown that periodontitis is associated with a higher risk for prostate cancer (14%; hazard ratio (HR) = 1.14, 95% confidence interval (CI) = 1.01–1.31, P = 0.042) (Lee et al., 2017). Another study on 5,199 patients with 7 years of follow-up even demonstrated an increase in the risk of cancer to 17% in subjects with active periodontal diseases. Furthermore the risk of prostate cancer is significantly enhanced among men with periodontal diseases (Guven et al., 2019). Increased levels of prostate specific antigen (PSA), a marker used for prostate cancer screening, were found in patients with chronic periodontal disease (Lee et al., 2017b). Additionally, significant higher PSA levels have been reported in periodontitis patients compared to patients without periodontitis, and PSA levels decreased after periodontal treatment (Joshi et al., 2010; Nabil F Bissada, 2015). All these findings show that periodontitis is associated with increased risks of prostate cancer, the second most common cancer in men. While patients with localized tumors mostly exhibit good long-term survival after treatment, mCRPC patients show worse survival outcomes (Klotz, 2000; de Bono et al., 2011).

The strength of this study is that it presents novel insights in a possible link between periodontitis and prostate cancer, two diseases that are very frequent. Components of the periodontal pathogen *P. gingivalis* that play an important role in the up-regulation of an immunomodulatory receptor, that is shown to be associated with growth and progression of a number of human cancers, were identified.

It also has to be given account for the fact that it is an *in vitro* study using a cell line. Thus, the results can only limitedly be translated in the *in vivo* situation.

In conclusion, this study provides novel evidence that viable and heat-killed *P. gingivalis* upregulates PD-L1 expression in prostate cancer cells and demonstrates that *P. gingivalis* membrane fractions are responsible for this PD-L1 induction. This study also shows the involvement of the NOD receptor signaling pathway during *P. gingivalis* infection. The demonstrated upregulation of PD-L1 may contribute to cancer immune evasion. *P. gingivalis* infection might be a risk factor for prostate cancer and that the ensuing chronic inflammation may contribute to tumor immune evasion affecting the tumor microenvironment. Chronic infection may thus modify the immune response and have an impact on the progression of prostate cancer.

## Data Availability Statement

The raw data supporting the conclusions of this article will be made available by the authors, without undue reservation.

## Author Contributions

SG supervised the experiments and wrote the manuscript. FWu performed the experiments. FWa and TD helped to supervise the project. FD helped to perform the experiments. JM supervisedthe project. All authors contributed to the article and approved the submitted version.

## Funding

The study was supported by a grant from the Chinese government.

## Conflict of Interest

The authors declare that the research was conducted in the absence of any commercial or financial relationships that could be construed as a potential conflict of interest.

## Publisher’s Note

All claims expressed in this article are solely those of the authors and do not necessarily represent those of their affiliated organizations, or those of the publisher, the editors and the reviewers. Any product that may be evaluated in this article, or claim that may be made by its manufacturer, is not guaranteed or endorsed by the publisher.
