# Supplementary material for: PD-L1 Up-Regulation in Prostate Cancer Cells by Porphyromonas gingivalis
Source: Front Cell Infect Microbiol. 2022 Jun 29;12:935806. doi: 10.3389/fcimb.2022.935806 (PMC9277116; doi:10.3389/fcimb.2022.935806)
Supplement: Supplementary file 1 [file Table_1.docx]

| **Sample** | **p-value (students t-test)** | **Corrected p-value (Bonferroni-Holms)** |
| --- | --- | --- |
| Prot. Neg – P.g. viable MOI 10 | 0.00716113 | 0.0214834 |
| Prot. Neg – P.g. viable MOI 100 | 0.002017883 | 0.0060536 |
| Prot. Neg – P.g. viable MOI 500 | 0.004102106 | 0.0123063 |
| Prot. Neg – P.g. hk MOI 10 | 6.10537E-06 | 0.0121871 |
| Prot. Neg – P.g. hk MOI 100 | 0.015749962 | 0.0472499 |
| Prot. Neg – P.g. hk MOI 500 | 0.000152346 | < 0.001 |
| Prot. Neg – TM | 0,000381679 | 0.0015267 |
| Prot. Neg – OM | 0,01282676 | 0.0256535 |
| Prot. Neg – Cyt | 0,577024688 | 0.5770247 |
| Prot. Neg – IFN-γ | 0,004465112 | 0.0133953 |
| Prot. TM – JNK 10µM | 0,076224 | 0.076224 |
| Prot. TM – JNK 50µM | 2.84187E-05 | < 0.001 |
| Prot. TM – JNK 100µM | 2.03903E-07 | < 0.001 |
| Prot. TM – RIP2 0.2µM | 0.085921197 | 0.0859211965745162 |
| Prot. TM – RIP2 5µM | 2.18213E-05 | < 0.001 |
| Prot. TM – RIP2 10µM | 0.001231797 | 0.0024636 |
| Prot. Neg – TM | 0.004270436 | 0.0128113 |
| Prot. Neg – ieDAP 10µg/ml | 0.202893631 | 0.4057873 |
| Prot. Neg – ieDAP 20g/ml | 0.218281626 | 0.4057873 |
| Prot. Neg – TM | 3.0787E-09 | < 0.001 |
| Prot. Neg – PGN | 9.35134E-08 | < 0.001 |
| RNA PD-L1 Neg - TM | 1,37107E-05 | < 0.001 |
| RNA RIP2 Neg - TM | 0.038025444 | 0.0380254 |
| RNA PD-L1 Neg - ieDAP | 0.036868566 | 0.0368686 |
| RNA RIP2 Neg - ieDAP | 1.23786E-06 | < 0.001 |
